# Stereoselective synthesis of whisky lactone isomers catalyzed by bacteria in the genus *Rhodococcus*

**DOI:** 10.3389/fmicb.2023.1117835

**Published:** 2023-01-19

**Authors:** Dawid Hernik, Francesco Gatti, Elisabetta Brenna, Ewa Szczepańska, Teresa Olejniczak, Filip Boratyński

**Affiliations:** ^1^Department of Food Chemistry and Biocatalysis, Wroclaw University of Environmental and Life Sciences, Wrocław, Poland; ^2^Dipartimento di Chimica, Materiali ed Ingegneria Chimica “Giulio Natta”, Politecnico di Milano, Milano, Italy

**Keywords:** biotransformation, fragrances, *Rhodococcus erythropolis*, lactones, oxidation, enantioselectivity

## Abstract

Whisky lactone is a naturally occurring fragrance compound in oak wood and is widely used as a sensory additive in food products. However, safe and efficient methods for the production of its individual enantiomers for applications in the food industry are lacking. The aim of this study was to develop an efficient and highly stereoselective process for the synthesis of individual enantiomeric forms of whisky lactones. The proposed three-step method involves (1) column chromatography separation of a diastereoisomeric mixture of whisky lactone, (2) chemical reduction of *cis-and trans*-whisky lactones to corresponding *syn-and anti*-diols, and (3) microbial oxidation of racemic diols to individual enantiomers of whisky lactone. Among various bacteria in the genera *Dietzia*, *Gordonia*, *Micrococcus*, *Rhodococcus,* and *Streptomyces*, *R. erythropolis* DSM44534 and *R. erythropolis* PCM2150 effectively oxidized *anti-and syn*-3-methyl-octane-1,4-diols (**1a-b**) to corresponding enantiomerically pure *cis-and trans*-whisky lactones, indicating high alcohol dehydrogenase activity. Bio-oxidation catalyzed by whole cells of these strains yielded enantiomerically pure isomers of *trans*-(+)-(4*S*,5*R*) (**2a**), *trans*-(−)-(4*R*,5*S*) (**2b**), and *cis*-(+)-(4*R*,5*R*) (**2d**) whisky lactones. The optical density of bacterial cultures and the impact of the use of acetone powders as catalysts on the course of the reaction were also evaluated. Finally, the application of *R. erythropolis* DSM44534 in the form of an acetone powder generated the enantiomerically enriched *cis*-(−)-(4*S*,5*S*)-isomer (**2c**) from the corresponding *syn*-diol (**1b**). The newly developed method provides an improved approach for the synthesis of chiral whisky lactones.

## 1. Introduction

Whisky lactone is a crucial ingredient in aged alcoholic beverages, such as whisky, cognac, and brandy (Maga, 1996) and a fragrance ingredient in various foods (e.g., sweet and baked foods) and beverages. It is also used as a repellent against mosquitoes and flies (Suzukt et al., 1992). Whisky lactone was first identified in 1970 by Suomalainen and Nykanen (Suomalainen and Nykanen, 1970) as a single compound in many alcohols in oak barrels; therefore, it is commonly named oak lactone. Masuda and Nishimura (Masuda and Nishimura, 1971) later discovered that two diastereoisomers can be isolated from oak wood species. Then, in 1989, Gunther and Mosandl separated four whisky lactone stereoisomers. Mixtures of isomers have been described as reminiscent of coconut, while *cis*-isomers have been characterized as woody and earthy and *trans*-isomers as celery-like. It should be noted that in nature, oak wood contains only *trans*-(+)-(4*S*,5*R*) and *cis*-(−)-(4*S*,5*S*) whisky lactone isomers (Abbott et al., 1995).

Several processes for *trans-and cis*-isomers of whisky lactone synthesis have been described (Ito et al., 1996; Brenna et al., 2001; Armstrong et al., 2009; Jiang et al., 2010; Pisani et al., 2012; Boratyński et al., 2013, 2014, 2018; Xie et al., 2017). However, these processes are based on multi-stage chemical synthesis using metal catalysts and organic solvents. Despite their wide use, metal-based catalysts are often harmful to the environment. A method for obtaining lactone stereoisomers with non-metallic catalysts (Xie et al., 2017) has also been described; however, the multistep approach was characterized by a relatively low conversion. Therefore, safe methods for the production of each stereoisomers of whisky lactone that fulfill green chemistry requirements are needed. Several biocatalytic pathways lead to optically active stereoisomers of whisky lactone. For example, alcohol dehydrogenase isolated from horse liver (HLADH) enantioselectively oxidizes racemic *syn-* and *anti*-3-methyloctane-1,4-diols (Boratyński et al., 2014). Another method uses whole cells of *Beauveria bassiana* AM278 and *Pycnidiella resinae* KCH50 for the lactonization of γ-oxo acids (Boratyński et al., 2013). Alternatively, the *trans*-(+)-(4*S*,5*R*) enantiomer of whisky lactone is obtained by reduction of the corresponding γ-oxo acids catalyzed by baker’s yeast (Brenna et al., 2001).

The chirality of chemical compounds is very important in biological processes. In the case of the production of chiral drugs, enantiomers may interact differently with individual metabolic systems (Beck, 2002). Enantiomers can also evoke different aroma sensations or have a different odor intensity (expressed as the odor threshold). Since the properties of individual enantiomers can vary substantially, it is important to develop methods for obtaining enantiomerically pure compounds. One such method is biotransformation involving whole microbial cells (bacteria, yeast, or fungi) or isolated enzymes (Nagy et al., 2006; Marie et al., 2011; Chreptowicz et al., 2016). This method can be used to generate optically active compounds that occur naturally and are difficult to obtain by chemical methods (Braga and Belo, 2016).

The genus *Rhodococcus* (phylum *Actinobacteria*) includes Gram-positive, non-motile aerobic bacteria (Alvarez, 2019). Bacteria in this genus have been isolated from soil, groundwater, marine sediments, and diseased and healthy animals and plants (Larkin et al., 2006). Only a few species are pathogenic, e.g., *R. equi* (a cause of foal pneumonia) and *R. fascians* (a cause of leafy gall disease). Various *Rhodococcus* strains have been used as biocatalysts for the degradation of natural organic compounds as well as xenobiotics (Kim et al., 2018). These bacteria show, *inter alia*, the ability to biodegrade short-and long-chain alkanes and aromatic, heterocyclic, and polycyclic compounds (Larkin et al., 2005). They are characterized by high metabolic diversity, indicating high tolerance against a wide range of substrates and solvents (Liang et al., 2019). For this reason, the use of *Rhodococcus* strains in the bioremediation of organic pollutants from petroleum, like *o*-xylene, has been investigated (Kim et al., 2002, 2010). The degradation of lignins *via R. jostii* RHA1 can lead to the production of vanillin, a valuable flavor compound (Ahmad et al., 2011). *Rhodococcus* members are also able to carry out the desulfurization reaction and therefore can degrade sulfur-containing compounds found in fossil fuels, like benzothiophene or dibenzothiophene (Khairy et al., 2015). *Rhodococcus* has a wide range of enzymatic activities and is therefore a biocatalyst of choice in various biotransformation processes involving alcohol dehydrogenases (ADHs), oxidases, monooxygenases, dioxygenases, reductases, etc. (Stampfer et al., 2002; Nikodinovic et al., 2006; Kim et al., 2013; Ewing et al., 2015; Nolte and Urlacher, 2015; Biermann et al., 2016; Müller et al., 2016; Wu and Li, 2018; Sheldon and Brady, 2019).

The aim of this study was to develop a biocatalytic method of obtaining industrially valuable optically active whisky lactones. Established methods are limited and usually characterized by low stereoselectivity. Herein, selected bacterial strains were tested for the bio-oxidation of *anti-* and *syn*-3-methyloctane-1,4-diols (1a-b). Two strains of *R. erythropolis* with high ADH activity stereoselectively catalyzed biotransformation, yielding highly enantioenriched whisky lactone isomers. The newly described method is a cost-efficient strategy for the asymmetric synthesis of each stereoisomer of whisky lactones.

## 2. Materials and methods

### 2.1. Microorganisms

*Dietzia maris* PCM2292, *Gordonia bronchialis* PCM2167*, Gordonia rubripertincta* PCM2144, *Micrococcus luteus* PCM525*, Rhodococcus coprophilus* PCM2174*, Rhodococcus erythropolis* PCM2150*, Rhodococcus rhodnii* PCM2157*, Rhodococcus rhodochrous* PCM909*, Rhodococcus ruber* PCM2166*, Rhodococcus ruber* PCM2171*, Rhodococcus ruber* PCM2216, and *Streptomyces griseus* subsp. *griseus* PCM2331 were purchased from the Polish Academy of Sciences*. Dietzia* sp. DSM44016 and *Rhodococcus erythropolis* DSM44534 were purchased from the German Collection of Microorganisms and Cell Cultures. Biocatalysts were maintained at 4°C on PCM medium agar slants and were then transferred into conical flasks with PCM medium containing sodium chlorine (6 g), glucose (20 g), casein (2 g), bacteriological peptone (10 g), and yeast extract (2 g) dissolved in distilled water (1 L) at 25°C and pH 5.5.

### 2.2. Materials

A diastereoisomeric mixture of whisky lactones, nicotinamide adenine dinucleotide (NAD^+^), nicotinamide adenine dinucleotide phosphate (NADP^+^), flavin mononucleotide (FMN), glutamate dehydrogenase (GDH), LiAlH_4_, and PCM medium ingredients was purchased from Sigma-Aldrich Chemical Co. (St. Louis, MO, USA).

### 2.3. Preparation of substrates for biotransformation

A commercially available diastereoisomeric mixture of *cis*/*trans*-whisky lactones was separated by column chromatography to obtain individual *trans* (0.430 g) and *cis* (0.500 g)-whisky lactones, which were subsequently chemically reduced to corresponding *anti*-(0.380 g) and *syn*-3-methyl-octane-1,4-diol (0.456 g) diols (**1a-b**). This procedure has been described by us in detail (Hernik et al., 2021). The NMR and IR spectra of lactones and diols are as follows:

*Trans*-whisky lactone (**2a-b**) ^1^H NMR (600 MHz, CDCl_3_) δ: 0.91 (t, J = 7.2 Hz, 3H, CH_3_-4′); 1.13 (d, J = 6.5 Hz, 3H, CH_3_-4); 1.32–1.42 (m, 3H, CH_2_-3′, one of CH_2_-2′); 1.50 (m, 1H, one of CH_2_-2′); 1.60 (m, 1H, one of CH_2_-1′); 1.68 (m, 1H, one of CH_2_-1′); 2.15–2.25 (m, 2H, one of CH_2_-3, H-4); 2.66 (m, 1H, one of CH_2_-3); 4.00 (td, J = 7.9, 4.0 Hz, 1H, H-5); ^13^C NMR (150 MHz, CDCl_3_): δ 13.89 (C-4′), 17.49 (CH_3_-4), 22.49 (C-3′), 27.85 (C-2′), 33.70 (C-1′), 36.08 (C-4), 37.13 (C-3), 87.46 (C-5), 176.61 (C-2); IR (film, cm^−1^): 1787 (s), 1222 (s), 1187 (s).

*Cis*-whisky lactone (**2c-d**) ^1^H NMR (600 MHz,CDCl_3_) δ: 0.91 (t, J = 7.3 Hz, 3H, CH_3_-4′); 1.00 (d, J = 7.0 Hz, 3H, CH_3_-4); 1.29–1.40 (m, 3H, CH_2_-3′, one of CH_2_-2′); 1.45–1.54 (m, 2H, one of CH_2_-2′, one of CH_2_-1′); 1.65 (m, 1H, one of CH_2_-1′); 2.18 (dd, J = 17.0, 4.0 Hz, 1H, one of CH_2_-3); 2.57 (m, 1H, H-4); 2.67 (dd, J = 17.0, 7.8 Hz, 1H, one of CH_2_-3); 4.42 (ddd, J = 10.1, 5.6, 4.1 Hz, 1H, H-5); ^13^C NMR (150 MHz, CDCl_3_): δ 13.82 (CH_3_-4), 13.90 (C-4′), 22.51 (C-3′), 28.03 (C-2′), 29.57 (C-4), 33.01 (C-1′), 37.56 (C-3), 83.70 (C-5), 176.94 (C-2); IR (film, cm^−1^): 1787 (s), 1,219 (m), 1,180 (s).

*Anti*-3-methyloctane-1,4-diol (**1a**) ^1^H NMR (600 MHz,CDCl_3_) δ: 0.87 (d, J = 6.8 Hz, 3H, CH_3_-3); 0.89 (t, J = 7.1 Hz, 3H, CH_3_-8); 1.22–1.36 (m, 3H, one of CH_2_-6, CH_2_-7); 1.38–1.46 (m, 3H, CH_2_-5, one of CH_2_-6); 1.50 (m, 1H, one of CH_2_-2); 1.67–1.77 (m, 2H, one of CH_2_-2, H-3); 2.81 i 3.00 (two s, 2H, 2xOH); 3.55 (m, 1H, H-4); 3.62 (ddd, J = 10.9, 7.1, 5.0 Hz, 1H, one of CH_2_-1); 3.73 (ddd, J = 10.9, 6.4, 5.0 Hz, 1H, one of CH_2_-1); ^13^C NMR (150 MHz, CDCl_3_) δ: 13.89 (CH_3_-3), 14.12 (C-8), 22.79 (C-7), 28.70 (C-6), 33.35 (C-5), 35.99 (C-3), 36.20 (C-2), 60.65 (C-1), 74.97 (C-4); IR (film, cm^−1^): 3342 (s), 1475 (m), 1,395 (m), 1065 (m), 1,018 (m).

*Syn*-3-methyloctane-1,4-diol (**1b**) ^1^H NMR (600 MHz, CDCl_3_) δ: 0,89 (t, J = 7.1 Hz, 3H, CH_3_-8); 0.92 (d, J = 6.8 Hz, 3H, CH_3_-3); 1.23–1.36 (m, 3H, one of CH_2_-6, CH_2_-7); 1.37–1.51 (m, 3H, CH_2_-5, one of CH_2_-6); 1.56 (m, 1H, one of CH_2_-2); 1.62–1.70 (m, 2H, one of CH_2_-2, H-3);3.13 (s, 2H, 2xOH); 3.38 (ddd, J = 8.4, 5.5, 3.3 Hz, 1H, H-4); 3.59 (ddd, J = 11.4, 6.9, 5.1 Hz, 1H, one of CH_2_-1); 3.72 (ddd, J = 11.4, 6.7, 5.0 Hz, 1H, one of CH_2_-1); ^13^C NMR (150 MHz, CDCl_3_) δ: 14.12 (C-8), 16.60 (CH3-3), 22.81 (C-7), 28.06 (C-6), 34.14 (C-5), 35.26 (C-2), 36.43 (C-3), 60.31 (C-1), 75.82 (C-4); IR (film, cm^−1^): 3333 (s), 1,480 (s), 1386 (s), 1069 (s), 1018 (s).

### 2.4. Preliminary screening-scale biotransformations in microtiter plates

Twenty-four well MTPs were sterilized at 121°C and 1 atm. Then, 4 mL of sterile PCM medium was added to each well of the MTP. Holes were inoculated with 0.2 mL of pre-prepared cultures of bacteria at OD_600_ = 0.3 and shaken (200 rpm) for 24 h at 22°C. Then, 0.002 g of the substrate (*anti*-3-methyloctane-1,4-diol (**1a**) or *syn*-3-methyloctane-1,4-diol (**1b**)) dissolved in 0.1 mL of acetone was added to every well. For simple extraction, ethyl acetate (0.7 mL) was added to the samples (1 mL) and shaken for 5 min at 200 rpm in 2 mL Eppendorf tubes. The organic phase was transferred to a vial and dehydrated by anhydrous MgSO_4_. Then, it was filtered through a paper filter to a GC vial. Biotransformation was controlled after 6, 24, and 48 h on the GC. Control experiments were also performed in which microorganisms were cultured on the medium without the addition of substrate to check metabolites.

### 2.5. Screening-scale biotransformations

Forty milliliters of PCM medium were added to 100 mL tapered flasks and then sterilized at 121°C at a pressure of 1 atm. The medium was inoculated with 0.5 mL of pre-culture of bacteria at OD_600_ = 0.3. The prepared bacterial cultures were placed for 3 days at 22°C and shaken at 150 rpm. Then, 0.01 g of the substrate (*anti*-3-methyloctane-1,4-diol (**1a**) or *syn*-3-methyloctane-1,4-diol (**1b**)) dissolved in 0.5 mL of acetone water was added to each of the flasks. For simple extraction, ethyl acetate (5 mL) was added to the samples (10 mL) and shaken for 5 min at 200 rpm in Falcon tubes. The organic phase was transferred to a vial and dehydrated by anhydrous MgSO_4_. Then, it was filtered through a filter paper to a GC vial. Biotransformation was controlled after 3 and 7 days on the GC.

### 2.6. Preparative biotransformations

Eighty milliliters of PCM medium were placed in 250 mL Erlenmeyer flask and sterilized at 121°C for 15 min. The medium was inoculated with 5 mL of preprepared cultures of bacteria at OD_600_ = 0.3. Erlenmeyer flasks with bacterial cultures were placed for 3 days at 22°C and shaken at 150 rpm. After incubation for 3 days, 0.05 g of the substrate (*anti*-3-methyloctane-1,4-diol (**1a**) or *syn*-3-methyloctane-1,4-diol (**1b**)) dissolved in 2 mL of acetone was added to the culture. Samples were extracted after 24, 48, and 72 h and evaluated by GC.

### 2.7. Preparation of acetone powders

Five hundred milliliters of a 3-day bacterial culture were centrifuged at 12,000× *g* for 10 min at 4°C and the medium was separated from the bacterial cells. Cells were suspended in acetone (−20°C), centrifuged at 12,000× *g* for 10 min at 4°C, and the acetone was removed. This process was repeated three times and finally the cells were dried for 1 h to obtain dry and non-sticky acetone powders.

### 2.8. Biotransformations with acetone powders

Twenty-four well MTPs were sterilized at 121°C at a pressure of 1 atm. Then, 0.1 g of acetone powder was added with 3 mL of phosphate buffer (pH = 8.0) to each well of the MTP. Into each well, 0.002 g of NAD^+^ or NADP^+^ as coenzymes and 0.004 g of FMN or 0.001 g GDH as coenzyme regeneration agents were added in 0.1 ml of phosphate buffer (pH = 8.0). Subsequently, 0,010 g of the substrate (*anti*-3-methyloctane-1,4-diol (**1a**) or *syn*-3-methyloctane-1,4-diol (**1b**)) dissolved in 0.1 mL of acetone was added to every well and the MTP was shaken (200 rpm) at 22°C. For simple extraction, ethyl acetate (0.7 mL) was added to the samples (1 mL) and shaken for 5 min at 200 rpm in 2 mL Eppendorf tubes. The organic phase was transferred to a vial and dehydrated by anhydrous MgSO_4_. Then, it was filtered through a filter paper to a GC vial. Biotransformation was controlled after 6, 18, 42, and 66 h on the GC.

### 2.9. Analytical procedure

The separation of the diastereoisomeric mixture of *cis/trans*-whisky lactones and chemical reduction of whisky lactones to corresponding diols were controlled by thin layer chromatography, using aluminum foil plates coated with silica gel. Compounds were detected by spraying the plates with 1% Ce(SO_4_)_2_ and 2% H_3_[P(Mo_3_O_10_)_4_] in 10% H_2_SO_4_. Gas chromatography (GC, FID, carrier gas H_2_) was carried out using the Agilent Technologies 7,890 N (GC System, Santa Clara, CA, USA). Enantiomeric excesses of the products were determined on the chiral column Cyclosil-B (30 m × 0.25 mm × 0.25 μm; Agilent Technologies) according to the following temperature program: 80°C, 160°C (3°C/min), 250°C (20°C/min) (3 min). Samples (2 μL) were injected at a split ratio of 9:1; the carrier gas flow rate was 1 ml/min. The total run time was 34.0 min. Retention times (t) were established as follows: *t_R_* = 20.74 min for *trans*-(+)-(4*S*,5*R*) (**2a**), *t_R_* = 21.05 min for *trans*-(−)-(4*R*,5*S*) (**2b**), *t_R_* = 22.42 min for *cis*-(−)-(4*S*,5*S*) (**2c**), *t_R_* = 22.54 min for *cis*-(+)-(4*R*,5*R*) (**2d**) (Supplementary Figure S1). The substrates were determined on the chiral column CP-Chirasil L-Val (25 m × 0.25 mm × 0.12 μm; Agilent Technologies) according to the following temperature program: 80°C, 165°C (3°C/min), 200°C (20°C/min) (1 min). Samples (2 μL) were injected with a split ratio of 9:1; the flow of carrier gas was 1 mL/min. The total run time was 31.0 min. Retention times (t_R_) were established as follows: t_R_ = 18.553 for *anti*-3-methyloctane-1,4-diol (**1a**), t_R_ = 18.630 min for *syn*-3-methyloctane-1,4-diol (**1b**). The structures of the compounds were confirmed on the basis of ^1^H NMR and ^13^C NMR, which were recorded for CDCl_3_ solutions using a Bruker Advance DRX 600 (600 MHz) spectrometer (Billerica, MA, USA). IR spectra were determined using the FTIR Thermo-Mattson IR 300 Spectrometer. Optical rotations were measured on a Jasco P-2000 Polarimeter.

### 2.10. Statistical analysis

All experiments were performed in triplicate, and mean values are presented. For all comparisons, differences were not significant, as determined by Student’s *t*-tests. Additionally, the standard deviations were calculated for the conversion rate and percentages of whisky lactone isomers are shown in the tables. Statistical analyses were performed using Past 4.02.

## 3. Results and discussion

**Scheme 1:**
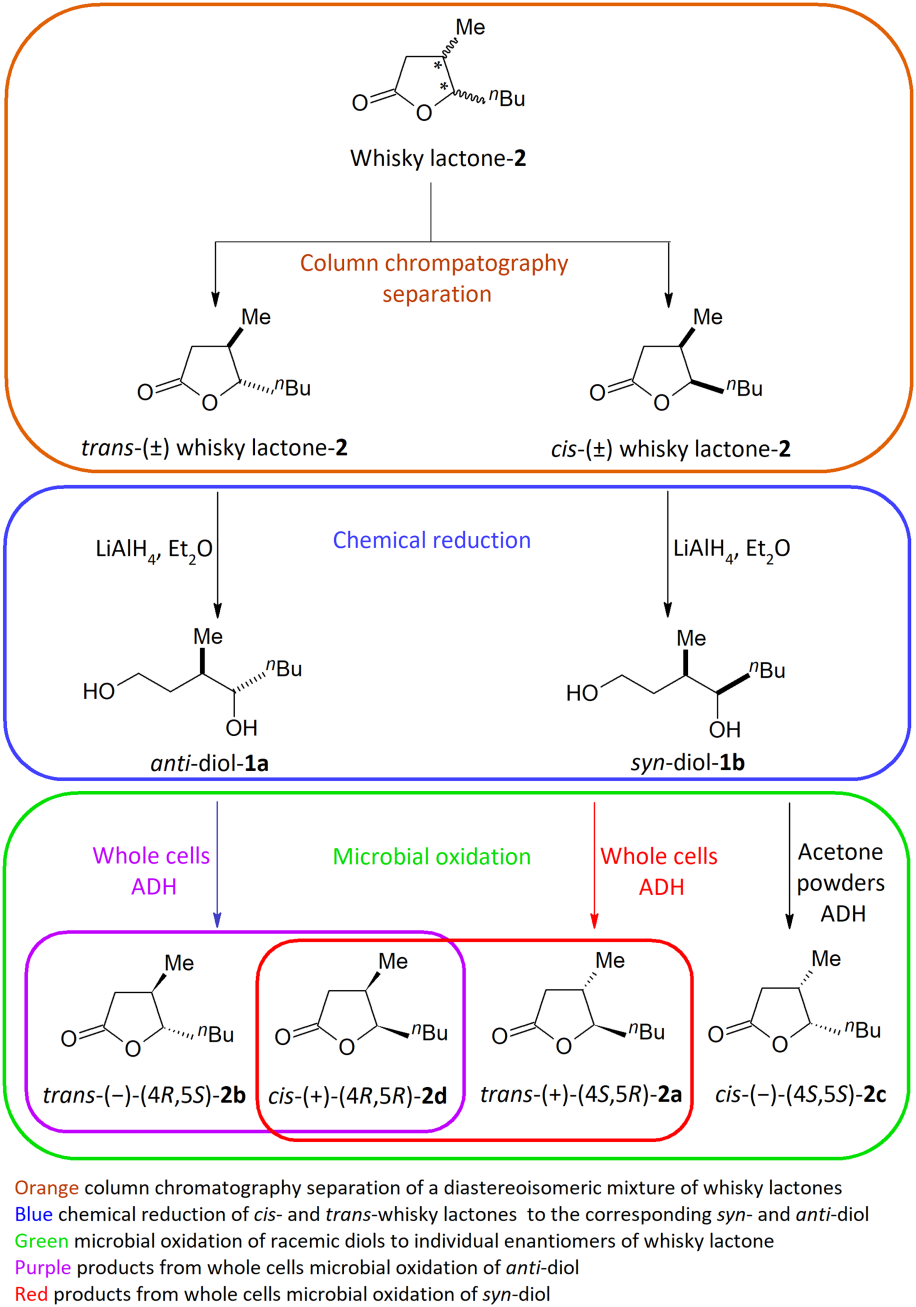
Overview of three steps in the chemo-enzymatic synthesis of individual whisky lactone enantiomers.

Fourteen bacterial strains in the genera *Dietzia*, *Gordonia*, *Micrococcus*, *Rhodococcus,* and *Streptomyces* were selected for screening-scale transformation in microtiter plates (MTPs) to obtain enantiomerically pure *trans-* and *cis*-whisky lactones (**2a–d**) from corresponding diols (**1a–b**). These microorganisms were chosen on the basis of our previous research focused on the sustainable management of oleo industry by-products in the microbial synthesis of whisky lactones (Hernik et al., 2021). Owing to the modest enantioselectivity and low yield in these previous semi-preparative-scale processes, our goal was to develop an efficient, and highly stereoselective process for the synthesis of individual enantiomeric forms of whisky lactones. Proposed by us three-step method involves (1) column chromatography separation of a diastereoisomeric mixture of whisky lactones, (2) chemical reduction of *trans-* and *cis*-whisky lactones (**2a-d**) (*de* > 99% determined by GC) with LiAlH_4_ to give the corresponding *syn-and anti*-diol, respectively (**1a-b**) (*de* > 99% determined by GC), and (3) microbial oxidation of racemic diols to individual enantiomers of whisky lactone (Scheme 1).

### 3.1. Preliminary screening-scale biotransformation in microtiter plates with *anti*-diol 1a

Initial biotransformations were carried out in MTPs. This allowed us a rapid selection of microorganisms with high ADH activity, responsible for the stereoselective oxidation of racemic diols to corresponding chiral lactones (Boratyński et al., 2013, 2016, 2020).

In oxidation of *anti*-3-methyloctane-1,4-diol (**1a**) the highest enantiomeric excess of *trans*-(+)-(4*S*,5*R*) isomer (**2a**) (*ee =* 96%) was detected after 24 h of transformation with *Dietzia* sp. DSM44016. In bio-oxidation with *R. erythropolis* DSM44534, *R. rhodnii* PCM2157, and *R. ruber* PCM 2166 for 24 h, enantiomeric excesses of *trans*-(+)-(4*S*,5*R*) isomer (**2a**) were lower (*ee* = 70–80%). It is worth mentioning that the opposite enantiomerically enriched *trans*-(−)-(4*R*, 5*S*) isomer (**2b**) (*ee* = 73%) was detected after 48 h of transformation with *R. erythropolis* PCM2150. The enantiomerically pure *cis*-(+)-(4*R*,5*R*) isomer (**2d**) (*ee* > 99%) was obtained by biotransformation with four strains: *Dietzia* sp. DSM44016, *R. erythropolis* DSM44534, *R. erythropolis* PCM2150, and *R. ruber* PCM2166. In bio-oxidation with the remaining strains, enantiomeric excesses of *trans-* and *cis*-whisky lactones were substantially lower than those for these four strains; using *S. griseus* subsp. *griseus* PCM2331, *D. maris* PCM2292, and *R. ruber* PCM2216, no conversion was observed (Table 1).

**Table 1:** Results of the microbial oxidation of *anti*-3-methyloctane-1,4-diol (1a) in MTPs.

Strain	Time [hours]	Conv. 1a [%]	Products			
			*Trans* [%]	*ee* [%]	*Cis* [%]	*ee* [%]
*Dietzia* sp. DSM44016	6	8 (±0.3)	–	–	8 (±0.8)	50 (+)
	24	100	72 (±1.2)	96 (+)	28 (±1.6)	99 (+)
	48	100	82 (±1.9)	67 (+)	18 (±0.7)	99 (+)
*Gordonia bronchialis* PCM2167	6	0	–	–	–	–
	24	45 (±1.9)	31 (±1.1)	50 (+)	14 (±0.9)	43 (+)
	48	100	64 (±2.1)	85 (+)	36 (±1.1)	71 (+)
*Gordonia rubripertincta* PCM2144	6	0	–	–	–	–
	24	43 (±1.3)	37 (±1.3)	–	6 (±0.4)	–
	48	89 (±2.0)	71 (±2.3)	67 (+)	18 (±0.9)	62 (+)
*Micrococcus luteus* PCM525	6	0	–	–	–	–
	24	0	–	–	–	–
	48	100	75 (±1.6)	65 (+)	25 (±1.2)	98 (+)
*Rhodococcus coprophilus* PCM2174	6	0	–	–	–	–
	24	60 (±0.9)	37 (±1.3)	5 (+)	23 (±1.0)	–
	48	100	62 (±0.9)	8 (+)	38 (±1.3)	3 (+)
*Rhodococcus erythropolis* DSM44534	6	27 (±1.0)	21 (±1.3)	50 (+)	6 (±0.2)	99 (+)
	24	89 (±1.2)	76 (±0.9)	71 (+)	13 (±0.4)	99 (+)
	48	100	72 (±1.7)	63 (+)	28 (±1.1)	99 (+)
*Rhodococcus erythropolis* PCM2150	6	0	–	–	–	–
	24	75 (±1.7)	65 (±2.1)	20 (−)	10 (±0.5)	99 (+)
	48	100	84 (±1.9)	73 (−)	16 (±0.7)	99 (+)
*Rhodococcus ruber* PCM2166	6	0	–	–	–	–
	24	13 (±0.5)	11 (±0.2)	70 (+)	2 (±0.1)	40 (+)
	48	100	80 (±2.3)	89 (+)	15 (±0.4)	99 (+)
*Rhodococcus ruber* PCM2171	6	0	–	–	–	–
	24	0	–	–	–	–
	48	72 (±1.1)	47 (±1.5)	17 (+)	25 (±0.5)	26 (+)
*Rhodococcus rhodnii* PCM2157	6	0	–	–	–	–
	24	52 (±1.3)	29 (±0.7)	80 (+)	23 (±0.3)	72 (+)
	48	93 (±2.5)	52 (±1.4)	82 (+)	41 (±1.9)	70 (+)
*Rhodococcus rhodochrous* PCM909	6	0	–	–	–	–
	24	80 (±1.4)	45 (±1.1)	50 (+)	35 (±0.9)	67 (+)
	48	81 (±1.6)	61 (±2.1)	61 (+)	20 (±0.2)	62 (+)

%, determined by CGC.

### 3.2. Preliminary screening-scale biotransformations in microtiter plates with *syn*-diol 1b

In biotransformations with *syn*-3-methyloctane-1,4-diol (**1b**) enantiomerically pure *trans*-(+)-(4*S*,5*R*) lactone (**2a**) was produced by *Dietzia* sp. DSM44016 and *R. erythropolis* PCM2150 after 24 h. On the other hand, enantiomerically pure *cis*-(+)-(4*R*,5*R*) whisky lactone (**2d**) was obtained with *R. erythropolis* DSM44534 and *R. erythropolis* PCM2150. For oxidation with *M. luteus* PCM525, *R. erythropolis* DSM44534, and *R. rhodnii* PCM2157, only *cis-*isomers of whisky lactone were formed. *M. luteus* PCM525 delivered *cis*-(−)-(4*S*,5*S*) (**2c**) with the highest enantiomeric excess (*ee* = 70%). In biotransformations with *S. griseus* subsp. *griseus* PCM2331, *R. coprophilus* PCM2174, *R. rhodochrous* PCM909, *R. ruber* PCM2171, and *G. rubripertincta* PCM2144, substantially lower enantiomeric excesses were observed than those achieved with other strains. No substrate conversion was detected using *D. maris* PCM2292 and *R. ruber* PCM2216 (Table 2).

**Table 2:** Results of the microbial oxidation of *syn*-3-methyloctane-1,4-diol (1b) in MTPs.

Strain	Time [hours]	Conv. 1b [%]	Products			
			*Trans* [%]	*ee* [%]	*Cis* [%]	*ee* [%]
*Dietzia* sp. DSM44016	6	0	–	–	–	–
	24	78 (±2.2)	15 (±1.0)	99 (+)	63 (±1.3)	64 (+)
	48	100	23 (±0.4)	98 (+)	77 (±1.5)	97 (+)
*Gordonia bronchialis* PCM2167	6	0	–	–	–	–
	24	79 (±1.5)	35 (±0.8)	62 (+)	44 (±0.5)	73 (+)
	48	100	45 (±1.5)	87 (+)	55 (±1.2)	92 (+)
*Gordonia rubripertincta* PCM2144	6	0	–	–	–	–
	24	50 (±0.9)	31 (±1.1)	5 (+)	19 (±0.4)	13 (+)
	48	74 (±1.5)	63 (±0.7)	58 (+)	11 (±1.1)	41 (+)
*Micrococcus luteus* PCM525	6	0	–	–	–	–
	24	85 (±2.1)	–	–	85 (±1.2)	70 (−)
	48	100	–	–	100 (±0.0)	20 (−)
*Rhodococcus coprophilus* PCM2174	6	0	–	–	–	–
	24	43 (±0.9)	24 (±1.1)	33 (+)	19 (±0.8)	17 (+)
	48	90 (±1.1)	47 (±2.1)	23 (+)	43 (±1.7)	7 (+)
*Rhodococcus erythropolis* DSM44534	6	40 (±1.1)	–	–	40 (±0.4)	90 (+)
	24	100	–	–	100	99 (+)
	48	100	–	–	100	99 (+)
*Rhodococcus erythropolis* PCM2150	6	11 (±0.2)	4 (±0.2)	–	7	–
	24	100	26 (±1.2)	99 (+)	74 (±1.5)	99 (+)
	48	100	11 (±0.9)	74 (+)	89 (±0.7)	99 (+)
*Rhodococcus ruber* PCM2166	6	0	–	–	–	–
	24	40 (±0.8)	–	–	40 (±0.9)	73 (+)
	48	89 (±1.3)	14 (±0.8)	98 (+)	75 (±1.0)	82 (+)
*Rhodococcus ruber* PCM2171	6	0	–	–	–	–
	24	32 (±0.4)	17 (±0.6)	–	15 (±0.8)	–
	48	80 (±1.5)	51 (±1.4)	19 (+)	29 (±1.1)	22 (+)
*Rhodococcus rhodnii* PCM2157	6	0	–	–	–	–
	24	52 (±2.0)	–	–	52 (±2.3)	67 (+)
	48	87 (±0.9)	–	–	87 (±1.9)	77 (+)
*Rhodococcus rhodochrous* PCM909	6	0	–	–	–	–
	24	60 (±1.7)	41 (±0.8)	35 (+)	19 (±0.3)	59 (+)
	48	100	63 (±1.2)	44 (+)	37 (±0.7)	63 (+)
*Streptomyces griseus* subsp. *griseus* PCM2331	6	0	–	–	–	–
	24	20 (±0.4)	8 (±0.2)	–	12 (±0.7)	10 (+)
	48	60 (±1.3)	23 (±0.8)	–	37 (±1.2)	9 (+)

%, determined by CGC.

Surprisingly, the enantiomerically pure or highly enriched *trans-and cis*-whisky lactones dominantly formed by selected microorganisms: *Dietzia* sp. DSM44016, *R. erythropolis* DSM44534, *R. erythropolis* PCM2150, and *R. ruber* PCM2166, indicate on dynamic kinetic resolution processes in performed biotransformations. Based on this preliminary results carried out in MTPs with *anti-and syn*-diols, aforementioned five microorganisms were selected for subsequent analyses.

### 3.3. Screening-scale biotransformations with *anti*-diol 1a

In the oxidation of *anti*-3-methyloctane-1,4-diol (**1a**) with *R. erythropolis* PCM2150, *R. erythropolis* DSM44534, and *Dietzia* sp. DSM44016, complete conversion (*conv.* = 100%) was observed after 6, 24, and 48 h, respectively. Oxidation with *R. erythropolis* PCM2150 afforded enantiomerically pure *trans*-(−)-(4*R*, 5*S*) (**2b**) (25%, *ee =* 99%) and *cis*-(+)-(4*R*,5*R*) (**2d**) (75%, *ee* = 99%) whisky lactone isomers after 48 h. In biotransformations conducted with *R. erythropolis* DSM44534, enantiomerically pure *trans*-(−)-(4*R*, 5*S*) (**2b**) (82%, *ee =* 99%) and *cis*-(+)-(4*R*,5*R*) (**2d**) (18%, *ee* = 99%) enantiomers were obtained after 48 h. After 72 h with the same strain, only the *trans*-(−)-(4*R*, 5*S*) (**2b**) isomer (100%, *ee* = 99%) was obtained. During transformation with *Dietzia* sp. DSM44016 after 72 h, enantiomerically enriched *trans*-(−)-(4*R*, 5*S*) (**2b**) (87%, *ee* = 54%) and enantiomerically pure *cis*-(+)-(4*R*,5*R*) (13%, *ee* = 99%) whisky lactone isomer (**2d**) were detected. In the course of biotransformation with *R. erythropolis* DSM44534 and *Dietzia* sp. DSM44016, *trans*-(+)-(4*S*,5*R*) (**2a**) enantiomer formation was relatively high, while *trans*-(−)-(4*R*, 5*S*) (**2b**) enantiomer became dominant over time. This trend was consistent with those in biotransformations in MTP, in which the formation of the *trans*-(+)-(4*S*, 5*R*) isomer (**2a**) in the initial stage was also observed. In addition, during biotransformation with *R. erythropolis* DSM44534 and *R. erythropolis* PCM2150, the same enantiomers of whisky lactone formed but in different amounts. Biotransformation with *R. ruber* PCM2166 showed a lower conversion rate (69%), and enantiomerically enriched *trans*-(+)-(4*S*,5*R*) (**2a**) (58%, *ee* = 64%) and *cis*-(+)-(4*R*,5*R*) (**2d**) (11%, *ee* = 72%) whisky lactones were acquired. The transformation with *R. ruber* PCM2157 showed very low conversion (5%) after 72 h (Table 3).

**Table 3:** Results of the biotransformation of *anti*-3-methyloctane-1,4-diol (1a).

Strain	Time [hours]	Conv. 1a [%]	Products			
			*Trans* [%]	*ee* [%]	*Cis* [%]	*ee* [%]
*Dietzia* sp. DSM44016	6	2 (±0.3)	-	-	2 (±0.1)	50 (+)
	24	25 (±1.3)	24 (±0.9)	92 (+)	1 (±0.1)	99 (+)
	48	100	95 (±1.0)	40 (+)	5 (±0.7)	99 (+)
	72	100	87 (±1.2)	54 (−)	13 (±0.9)	99 (+)
*Rhodococcus erythropolis* DSM44534	6	33 (±1.0)	29 (±0.8)	62 (+)	4 (±0.4)	99 (+)
	24	100	87 (±1.1)	30 (−)	13 (±0.7)	99 (+)
	48	100	82 (±1.3)	99 (−)	18 (±0.5)	99 (+)
	72	100	100	99 (−)	-	-
*Rhodococcus erythropolis* PCM2150	6	100	96 (±1.0)	0	4 (±0.7)	99 (+)
	24	100	47 (±0.3)	88 (−)	53 (±1.2)	99 (+)
	48	100	25 (±1.1)	99 (−)	75 (±1.8)	99 (+)
	72	100	22 (±0.6)	99 (−)	78 (±1.1)	99 (+)
*Rhodococcus ruber* PCM2166	6	0	-	-	-	-
	24	13 (±0.3)	11 (±0.9)	70 (+)	2 (±0.2)	40 (+)
	48	38 (±0.7)	34 (±0.5)	64 (+)	4 (±0.7)	75 (+)
	72	69 (±1.4)	58 (±1.6)	64 (+)	11 (±0.5)	72 (+)

%, determined by CGC.

### 3.4. Screening-scale biotransformation with *syn*-diol 1b

During the biotransformation of *syn*-3-methyloctane-1,4-diol (**1b**) with *R. erythropolis* DSM2150, *R. erythropolis* DSM44534, and *Dietzia* sp. DSM44016, the conversion rate was analogous to those for the transformation of *anti*-diol (**1a**). With *R. erythropolis* DSM2150, after 24 h, enantiomerically pure *trans*-(+)-(4*S*, 5*R*) (**2a**) (31%, *ee* = 99%) and *cis*-(+)-(4*R*,5*R*) (**2d**) (69%, *ee* = 99%) enantiomers of whisky lactone were obtained. After 48 h with the same strain, only the *cis*-(+)-(4*R*,5*R*) (**2d**) (100%, *ee* = 99%) isomer was acquired. In bio-oxidation with *R. erythropolis* DSM44534, after 24 h, only *cis*-(+)-(4*R*,5*R*) (**2d**) (100%, *ee* = 99%) whisky lactone was obtained. With *Dietzia* sp. DSM44016, after 48 h, enantiomerically enriched *trans*-(+)-(4*S*, 5*R*) (**2a**) (34%, *ee* = 94%) and *cis*-(+)-(4*R*,5*R*) (**2d**) (66%, *ee* = 97%) isomers formed. In biotransformation with *R. ruber* PCM2166, after 72 h, enantiomerically pure *trans*-(+)-(4*S*, 5*R*) (**2a**) enantiomer (11%, *ee* = 99%) and enantiomerically enriched *cis*-(+)-(4*R*,5*R*) (**2d**) (66%, *ee* = 97%) whisky lactone were obtained. Biotransformation with *M. luteus* PCM525 and *R. ruber* PCM2157 showed low conversion rates, while no conversion was observed with *G. bronchialis* PCM2167 (Table 4).

**Table 4:** Results of the biotransformation of *syn*-3-methyloctane-1,4-diol (1b).

Strain	Time [hours]	Conv. 1b [%]	Products			
			*Trans* [%]	*ee* [%]	*Cis* [%]	*ee* [%]
*Dietzia* sp. DSM44016	6	12 (±0.6)	–	–	12 (±0.3)	92 (+)
	24	67 (±1.6)	9 (±0.5)	99 (+)	58 (±1.7)	56 (+)
	48	100	34 (±1.4)	94 (+)	66 (±2.3)	97 (+)
	72	100	18 (±0.9)	94 (+)	82 (±2.5)	99 (+)
*Rhodococcus erythropolis* DSM44534	6	37 (±0.9)	–	–	37 (±1.0)	84 (+)
	24	100	–	–	100	99 (+)
	48	100	–	–	–	–
	72	100	–	–	–	–
*Rhodococcus erythropolis* PCM2150	6	100	7 (±0.2)	99 (+)	93 (±1.1)	34 (+)
	24	100	31 (±0.5)	99 (+)	69 (±1.4)	99 (+)
	48	100	0	–	100	99 (+)
	72	100	0	–	100	99 (+)
*Rhodococcus ruber* PCM2166	6	0	–	–	–	–
	24	9 (±0.8)	–	–	9 (±0.6)	68 (+)
	48	26 (±1.2)	2 (±0.1)	99 (+)	24 (±0.9)	70 (+)
	72	66 (±1.5)	11 (±0.7)	99 (+)	55 (±1.3)	68 (+)

%, determined by CGC.

Based on preliminary screening experiments for the preparative biotransformation of *anti-* and *syn-3-*methyl-octane-1,4-diols (**1a–b**) *R. erythropolis* DSM2150 and *R. erythropolis* DSM44534 were selected.

### 3.5. Preparative biotransformation with anti-diol 1a

The effect of the biomass concentration based on optical density (OD_600_ = 0.3, 0.5, and 1.0) during the course of biotransformation was evaluated. In a comparison of results obtained after 24, 48, and 72 h, no significant differences were detected in the enantiomeric excess of lactones and the conversion of diols. For this reason, preparative biotransformations were performed at OD_600_ = 1.0.

During biotransformation with *anti*-3-methyloctane-1,4-diol (**1a**), the time needed for conversion was substantially longer than that for biotransformation at a smaller scale. Moreover, the formation of byproducts was observed. In bio-oxidation with *R. erythropolis* DSM44534, after 144 h, enantiomerically pure *trans*-(−)-(4*R*,5*S*) (**2b**) ( ${\left[\alpha \right]}_{D}^{20}$ = −96.3 (c = 0.25, CH_3_OH, *ee =* 99%, *yield* = 22%); ref. ${\left[\alpha \right]}_{D}^{20}$ = −97.0 (c = 0.34, CH_3_OH, *ee* = 99%) (Wilkinson et al., 2004)) and *cis*-(+)-(4*R*,5*R*) (**2d**) ( ${\left[\alpha \right]}_{D}^{20}$ = +79.4 (c = 0.2, CH_3_OH, *ee* = 99%, *yield =* 8%); ref. ${\left[\alpha \right]}_{D}^{20}$ = +79.0 (c = 0.5, CH_3_OH, *ee* = 99%) (Wilkinson et al., 2004)) whisky lactones were detected (Table 5).

**Table 5:** Results of microbial oxidation of *anti*-3-methyloctane-1,4-diol (1a).

Strain	Time [hours]	Conv. 1a [%]	Products				
			*Trans* [%]	*ee* [%]	*Cis* [%]	*ee* [%]	Byproducts
*Rhodococcus erythropolis* DSM44534	96	100	92 (±1.2)	0	5 (±0.4)	99 (+)	2
	120	100	87 (±0.9)	0	5 (±0.4)	99 (+)	8
	144	100	37 (±0.5)	99 (−)	13 (±0.8)	99 (+)	55
*Rhodococcus erythropolis* PCM2150	96	100	100	5 (+)	–	–	0
	120	100	94 (±1.0)	5 (+)	6 (±0.7)	99 (+)	0
	144	100	0	0	0	0	100

%, determined by CGC.

### 3.6. Preparative biotransformation with *syn*-diol 1b

The conversion of *syn*-3-methyloctane-1,4-diol (**1b**) was fastest during bio-oxidation with *R. erythropolis* PCM2150 (after 18 h). With *R. erythropolis* DSM44534, complete conversion required 42 h. As a result of biotransformation catalyzed by *R. erythropolis* PCM2150, after 42 h, enantiomerically enriched *trans*-(+)-(4*S*,5*R*) (**2a**) ( ${\left[\alpha \right]}_{D}^{20}$ = +98.1 (c = 0.2, CH_3_OH, *ee =* 97%, *yield* = 14%); ref. ${\left[\alpha \right]}_{D}^{20}$ = +97.0 (c = 0.34, CH_3_OH, *ee* = 99%) (Wilkinson et al., 2004)) and optically pure *cis*-(+)-(4*R*,5*R*) (**2d**) ( ${\left[\alpha \right]}_{D}^{20}$ = +78.1 (c = 0.15, CH_3_OH, *ee* = 99%, *yield =* 60%); ref. ${\left[\alpha \right]}_{D}^{20}$ = +79.0 (c = 0.5, CH_3_OH, *ee* = 99%) (Wilkinson et al., 2004)) whisky lactones were obtained. During bio-oxidation with *R. erythropolis* PCM44534, after 42 h, enantiomerically enriched *trans*-(+)-(4*S*,5*R*) (**2a**) ( ${\left[\alpha \right]}_{D}^{20}$ = +94.8 (c = 0.1, CH_3_OH, *ee =* 90%, *yield* = 28%); ref. ${\left[\alpha \right]}_{D}^{20}$ = +97.0 (c = 0.34, CH_3_OH, *ee* = 99%) and pure *cis*-(+)-(4*R*,5*R*) (**2d**) ( ${\left[\alpha \right]}_{D}^{20}$ = +79.9 (c = 0.2, CH_3_OH, *ee* = 99%, *yield =* 40%); ref. ${\left[\alpha \right]}_{D}^{20}$ = +79.0 (c = 0.5, CH_3_OH, *ee* = 99%) (Wilkinson et al., 2004)) whisky lactones were obtained (Table 6).

**Table 6:** Results of the microbial oxidation of *syn*-3-methyloctane-1,4-diol (1b).

Strain	Time [hours]	Conv. 1b [%]	Products				
			*Trans* [%]	*ee* [%]	*Cis* [%]	*ee* [%]	Byproducts
*Rhodococcus erythropolis* DSM44534	18	77 (±1.0)	27 (±0.4)	18 (+)	50 (±1.1)	99 (+)	0
	42	100	37 (±1.1)	90 (+)	47 (±0.6)	99 (+)	16
*Rhodococcus erythropolis* PCM2150	18	100	23 (±0.7)	99 (+)	77 (±1.2)	58 (+)	0
	42	100	53 (±1.3)	97 (+)	47 (±0.9)	99 (+)	0

%, determined by CGC.

By comparing the results from the submerged preparative biotransformations described herein with our previous results obtained by solid-state fermentation (Boratyński et al., 2020), processes conducted in the SmF were characterized by the substantially greater and faster conversion of diols to corresponding whisky lactones. Moreover, the bio-oxidation carried out in SmF afforded enantiomerically pure lactones on a preparative scale, which could not be obtained in preparative SSF biotransformations. Among fourteen bacteria tested, bio-oxidation by *R. erythropolis* DSM44534 and *R. erythropolis* PCM2150 showed the highest efficiency and stereoselectivity, yielding *trans*-(+)-(4*S*,5*R*) (**2a**), *trans*-(−)-(4*R*,5*S*) (**2b**) and *cis*-(+)-(4*R*,5*R*) (**2d**) whisky lactones.

In our previous study, whisky lactone enantiomers were obtained by the microbial whole-cell reduction of γ-oxoacids (Boratyński et al., 2013). The *trans*-(+)-(4*S*,5*R*) (**2a**) enantiomer was obtained (*ee* = 99%) as the only product of the biotransformation catalyzed by *Didymosphaeria igniaria* KCH6651, *Laetiporus sulphurens* AM525, *Chaetomium* sp. KCH6670, and *Saccharomyces cerevisiae* AM464. However, during the biotransformation of the same γ-oxoacid by *Beauveria bassiana* AM278 and *Pycnidiella resinae* KCH50, a mixture of *trans*-(+)-(4*S*, 5*R*) (**2a**) (*ee* = 99%) and *cis*-(−)-(4*S*,5*S*) (**2c**) (*ee* = 45–77%) isomers was obtained. During enzymatic reactions catalyzed by the alcohol dehydrogenases HLADH and PADH I, enantiomerically enriched *trans*-(−)-(4*R*, 5*S*) (**2b**) and *cis*-(+)-(4*R*,5*R*) (**2d**) isomers were obtained (*ee* = 27–82%). In a previously described method of obtaining the *trans*-(+)-(4*S*, 5*R*) (**2a**) enantiomer (*ee* = 99%) by lactonization biocatalyzed by baker’s yeast, the efficiency on a preparative scale was 38% (Brenna et al., 2001). Compared to these results, *trans*-(+)-(4*S*,5*R*) (**2a**), *trans*-(−)-(4*R*,5*S*) (**2b**), and *cis*-(+)-(4*R*,5*R*) (**2d**) enantiomers of whisky lactone (*ee* = 97–99%) were obtained by the newly developed approach.

### 3.7. Screening-scale biotransformation with acetone powders in microtiter plates with anti and *syn*-diols 1a-b

Since the enantiomerically pure *cis*-(−)-(4*S*,5*S*) (**2c**) whisky lactone was not obtained, oxidation was carried out with acetone powders prepared from selected bacteria (*Dietzia* sp. DSM44016, *R. erythropolis* DSM44534, and *R. erythropolis* PCM2150). For enzymatic transformation, NAD^+^ and NADP^+^ as coenzymes and FMN and GDH for coenzyme regeneration were selected. Only NADP^+^ and GDH were suitable for biotransformations (Schenkels and Duine, 2000).

In all biotransformations of *anti*-3-methyloctane-1,4-diol (**1a**), only *trans*-whisky lactone isomers formed. In bio-oxidation with acetone powders from *Dietzia* sp. DSM44016, after 66 h, *trans*-(+)-(4*S*,5*R*) (**2a**) whisky lactone was detected (*ee* = 74%) with 100% conversion. Transformation with acetone powder from *R. erythropolis* DSM44534 after 42 h led to the *trans-*whisky lactone isomer *trans*-(−)-(4*R*,5*S*) (**2b**) (*ee* = 87%) with 85% conversion. It is worth noting that when using acetone powders from *Dietzia* sp. DSM44016 and *R. erythropolis* PCM2150, during the biotransformation of *anti*-diol (1a), an opposite *trans*-(+)-(4*S*,5*R*) (**2a**) isomer formed in the screening-scale whole-cell biotransformations. In transformations of *syn*-3-methyloctane-1,4-diol (**1a**) with acetone powders from *Dietzia* sp. DSM44016, *R. erythropolis* DSM44534, and *R. erythropolis* PCM2150, conversion was complete after 66 h. Oxidation with *R. erythropolis* DSM44534 acetone powders after 42 h afforded the highest enantiomeric excess (*ee* = 86%) of *cis*-(−)-(4*S*,5*S*) (**2c**) isomer, instead of *cis*-(+)-(4*R*,5*R*) (**2d**), which was obtained in whole-cell biotransformations with these bacteria. Biotransformations with acetone powders were characterized by lower and slower conversion than those in screening-scale bio-oxidation involving whole cells from the same strains. Additionally, in transformations with acetone powders, only one isomer always formed; when *anti*-diol (**1a**) was added as substrate, *trans*-whisky lactone formed, while *syn*-diol (**1b**) produced *cis*-whisky lactone. The application of acetone powders in biotransformations is often used to increase the stability of enzymes and improve enantioselectivity. Additionally, these biocatalysts frequently increase the yield and benefit from simple storage and use. For instance, the use of acetone powders from *Geotrichum candidum* by Nakamura and Madsuda (Nakamura and Matsuda, 1998) increased the enantioselectivity and efficiency of the reduction of ketones to alcohols and generated products with the opposite configuration. However, we did not observe a benefit of this biocatalyst over whole cell oxidation.

## 4. Conclusion

A chemo-enzymatic three-step method for obtaining whisky lactone isomers was developed. This method combined the separation of a diastereoisomeric mixture of whisky lactone isomers by column chromatography followed by chemical reduction to corresponding racemic diols. The latter are submitted to microbial oxidation to obtain each stereoisomer of whisky lactone. Among bacteria from different genera, *R. erythropolis* DSM44534 and *R. erythropolis* PCM2150 effectively oxidized *anti-* and *syn*-3-methyloctane-1,4-diols (**1a-b**) to corresponding whisky lactones, indicating high ADH activity. Bio-oxidation carried out on a preparative scale yielded enantiomerically pure isomers of *trans*-(+)-(4*S*,5*R*) (**2a**), *trans*-(−)-(4*R*,5*S*) (**2b**) and *cis*-(+)-(4*R*,5*R*) (**2d**) whisky lactones. In addition, it was developed that acetone powders prepared from selected bacteria could be used to generate enantiomerically enriched *cis*-(−)-(4*S*,5*S*) (**2c**) whisky lactone isomers, although the reactions were characterized by lower conversion. Based on the obtained results, it was noticed that the dynamic kinetic resolution processes are probably involved in the described whole cells transformations. Therefore, further studies are currently ongoing on wider portfolio of substrates to confirm the mechanism that occurs during this process.

## Data availability statement

The original contributions presented in the study are included in the article/Supplementary material, further inquiries can be directed to the corresponding authors.

## Author contributions

DH and FB: conceptualization, formal analysis, methodology, resources, and writing – original draft. DH: funding acquisition and investigation. FB and EB: supervision. DH and ES: visualization. TO, EB, and FG: writing – review and editing. All authors have read and agreed to the published version of the manuscript.

## Funding

This research and APC were funded by the project “UPWR 2.0: International and Interdisciplinary Program of Development of Wrocław University of Environmental and Life Sciences,” co-financed by the European Social Fund under the Operational Program Knowledge Education Development, under contract No. POWR.03.05.00-00-Z062/18 of 4 June 2019.

## Conflict of interest

The authors declare that the research was conducted in the absence of any commercial or financial relationships that could be construed as a potential conflict of interest.

## Publisher’s note

All claims expressed in this article are solely those of the authors and do not necessarily represent those of their affiliated organizations, or those of the publisher, the editors and the reviewers. Any product that may be evaluated in this article, or claim that may be made by its manufacturer, is not guaranteed or endorsed by the publisher.
