# Supplementary material for: Stereoselective synthesis of whisky lactone isomers catalyzed by bacteria in the genus Rhodococcus
Source: Front Microbiol. 2023 Jan 19;14:1117835. doi: 10.3389/fmicb.2023.1117835 (PMC9893411; doi:10.3389/fmicb.2023.1117835)
Supplement: Supplementary file 1 [file Data_Sheet_1.pdf]

Supplementary Material

Stereoselective synthesis of whisky lactone isomers catalyzed by bacteria in the genus *Rhodococcus*

Dawid Hernik[1*], Francesco Gatti[2], Elisabetta Brenna[2], Ewa Szczepańska[1], Teresa Olejniczak[1], Filip Boratyński[1*]

*** Correspondence:** Corresponding Authors:

dawid.hernik@upwr.edu.pl; filip.boratynski@upwr.edu.pl

1 Supplementary Data

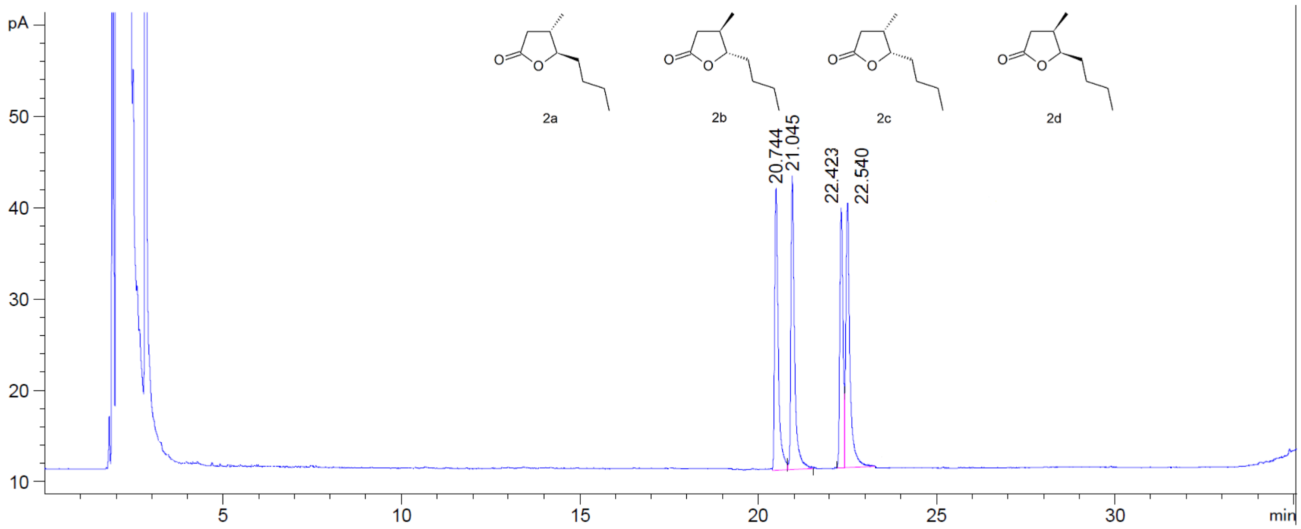

Figure S1. Chromatogram of the mixture of *trans* and *cis* whisky lactone

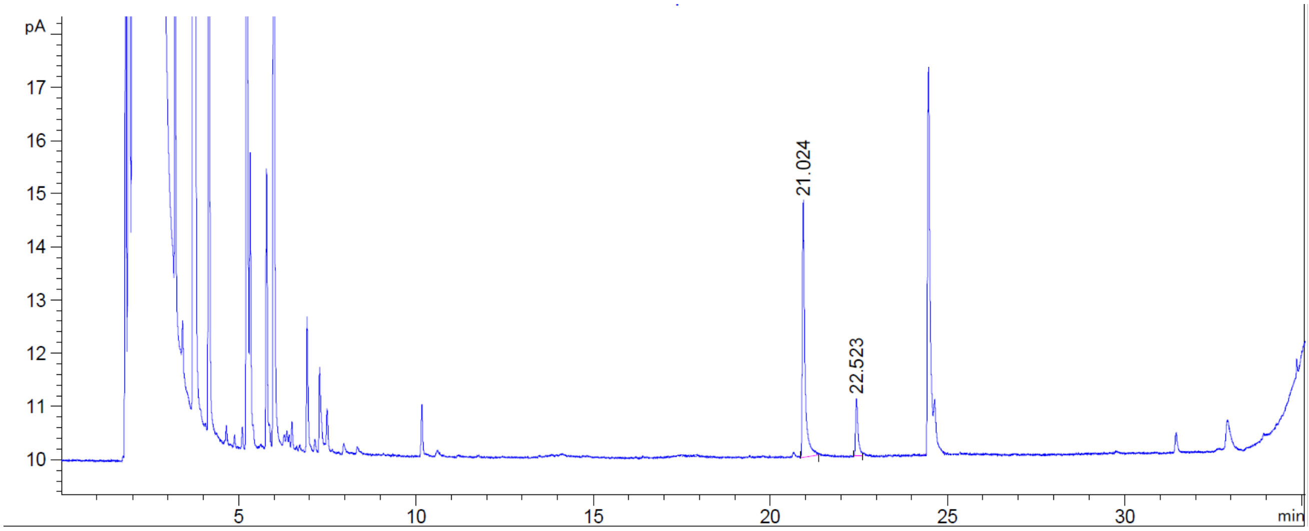

Figure S2. Chromatogram of the preparative scale oxidation of *anti*-3-methyloctane-1,4-diol with *R. erythropolis* DSM44534 after 144 hours.

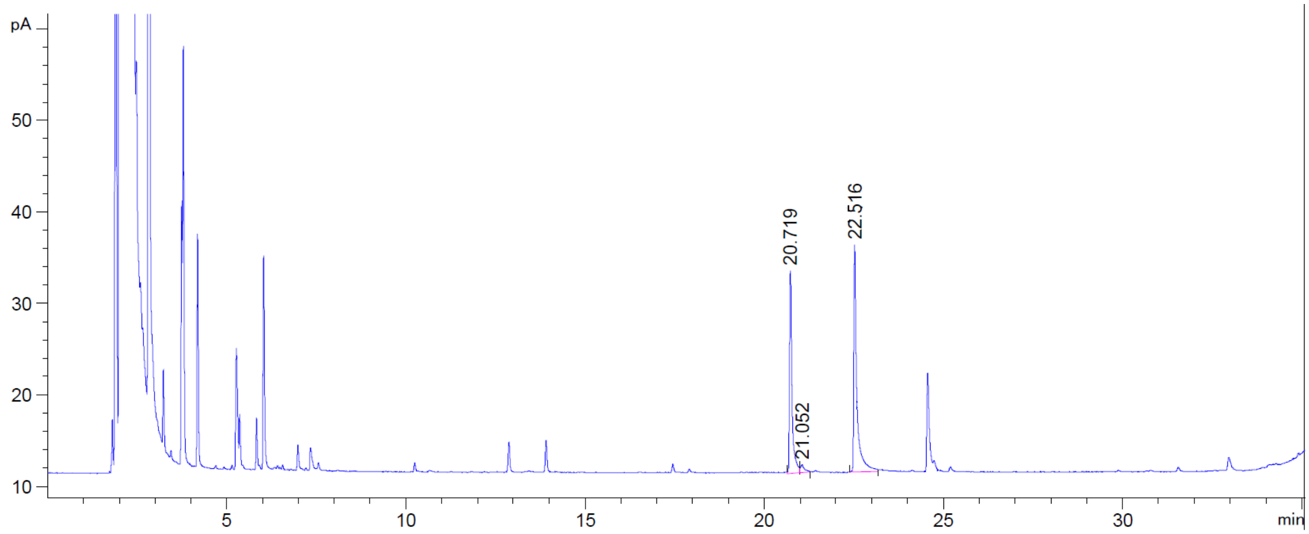

Figure S3. Chromatogram of the preparative scale oxidation of *syn*-3-methyloctane-1,4-diol with *R. erythropolis* DSM44534 after 42 hours.

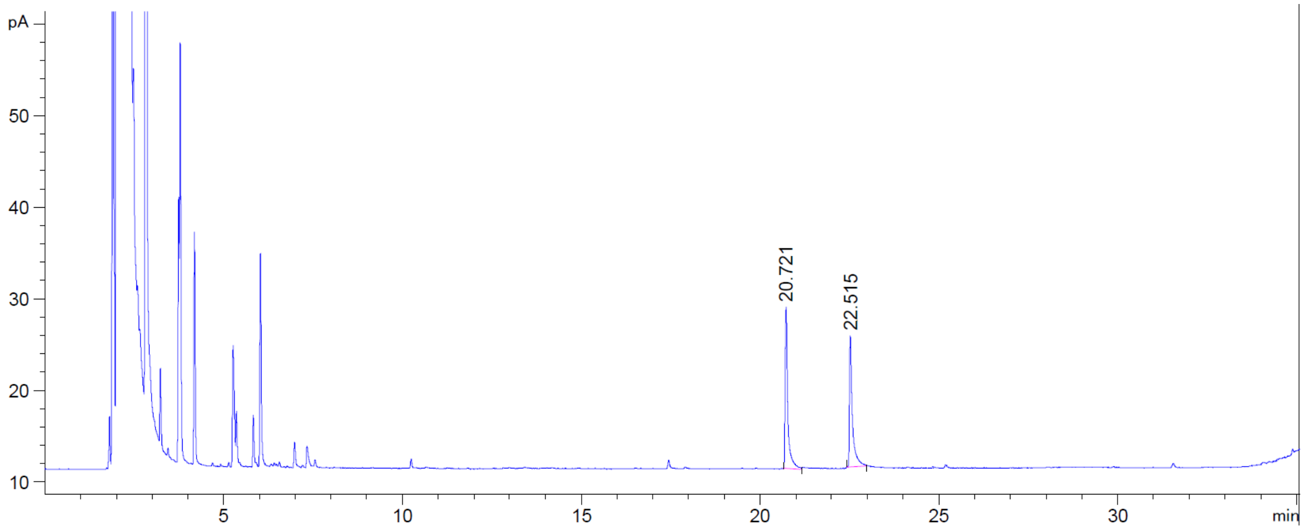

Figure S4. Chromatogram of the preparative scale oxidation of *syn*-3-methyloctane-1,4-diol with *R. erythropolis* PCM2150 after 42 hours.

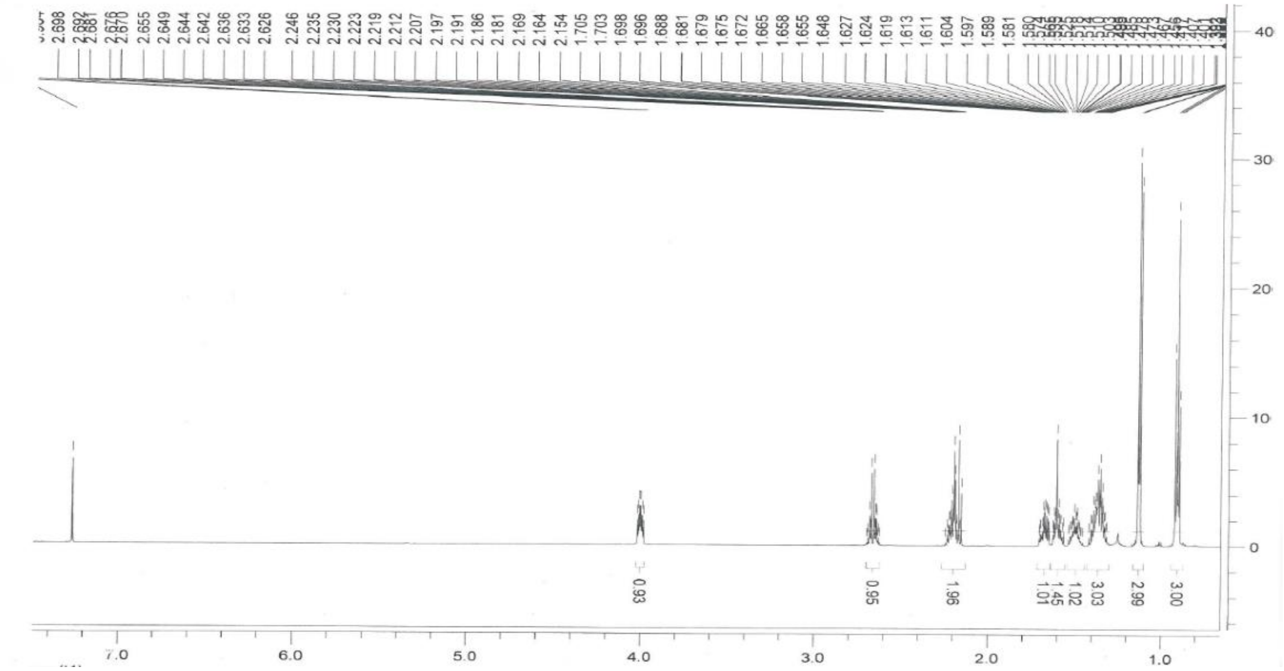

Figure S5. H[1] NMR spectrum of *trans*-whisky lactone

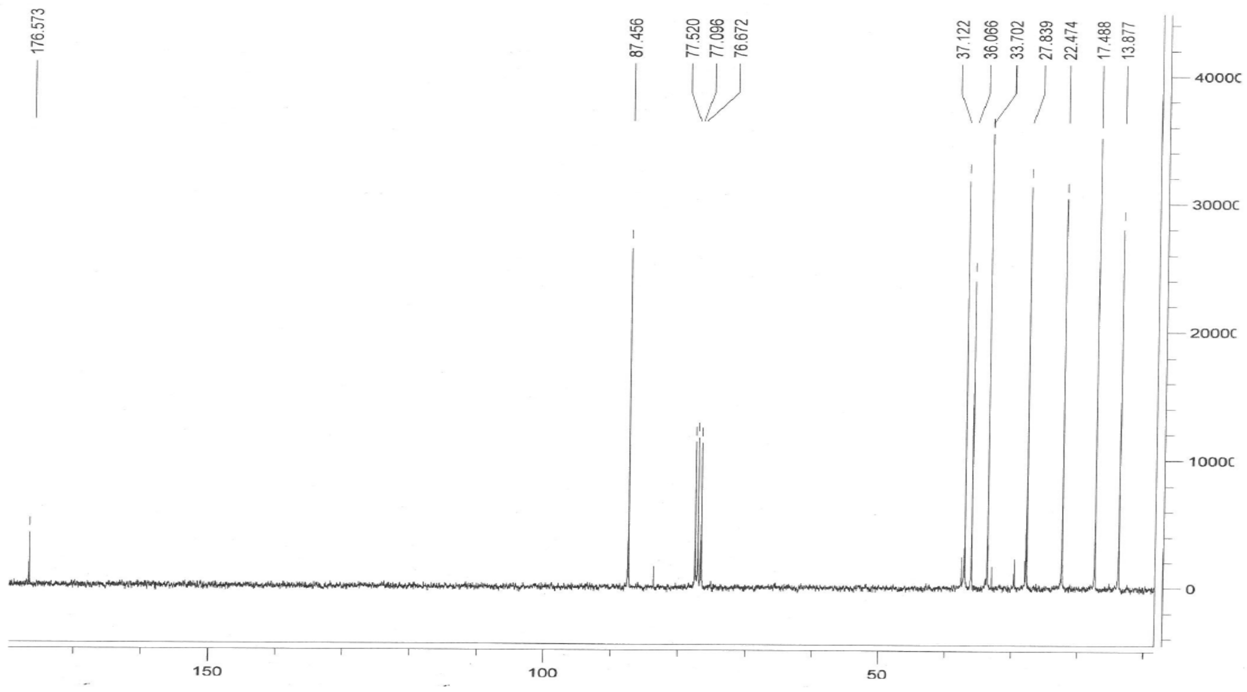

Figure S6. C^{13} NMR spectrum of *trans*-whisky lactone

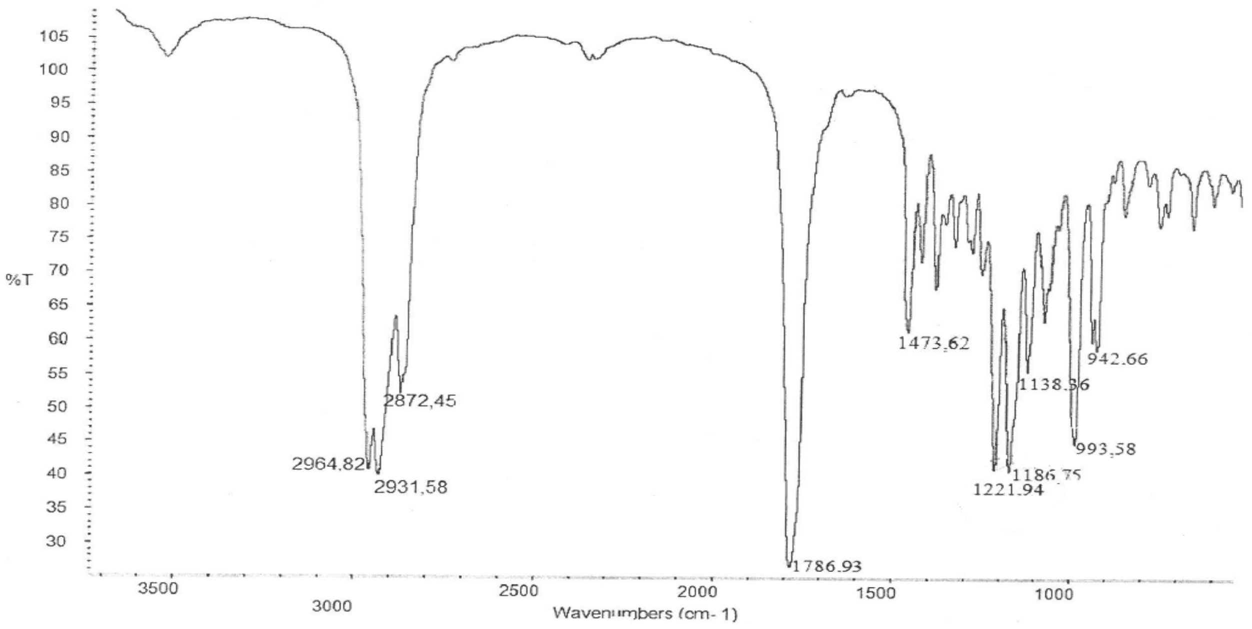

Figure S7. IR spectrum of *trans*-whisky lactone

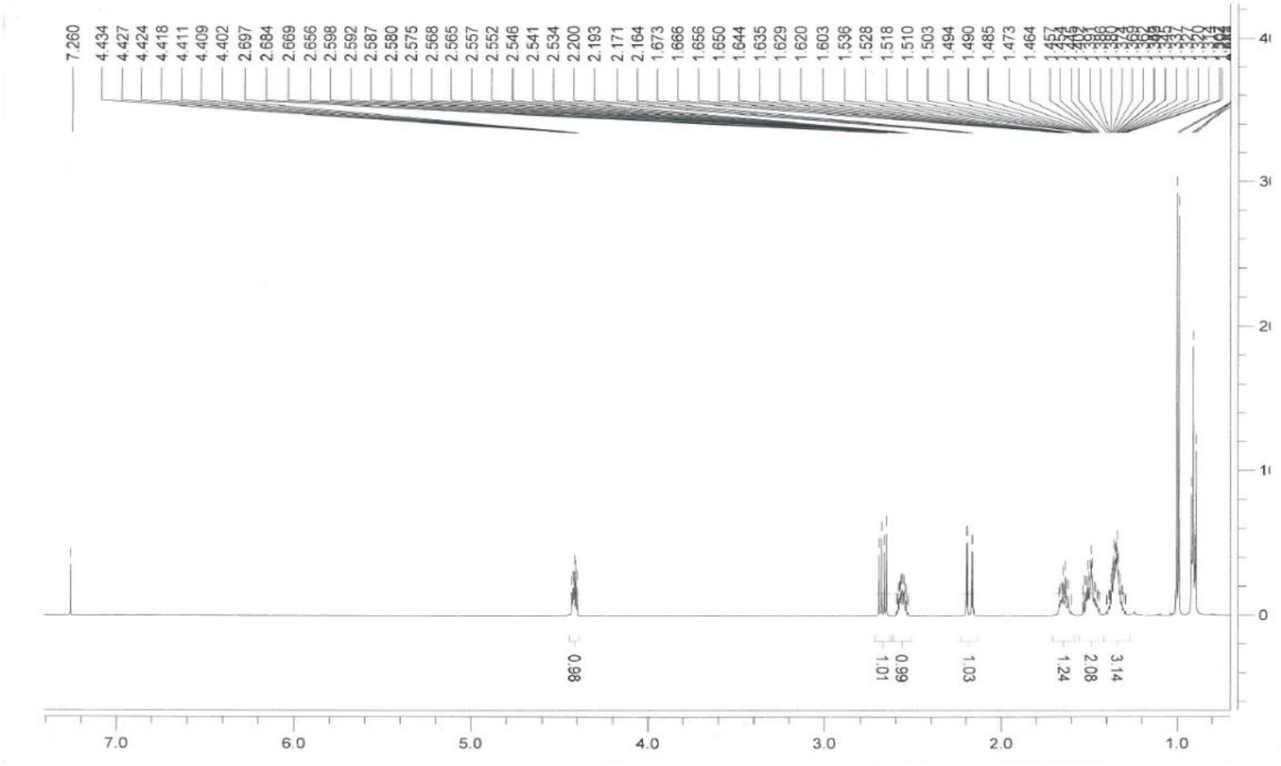

Figure S8. H^1 NMR spectrum of *cis*-whisky lactone

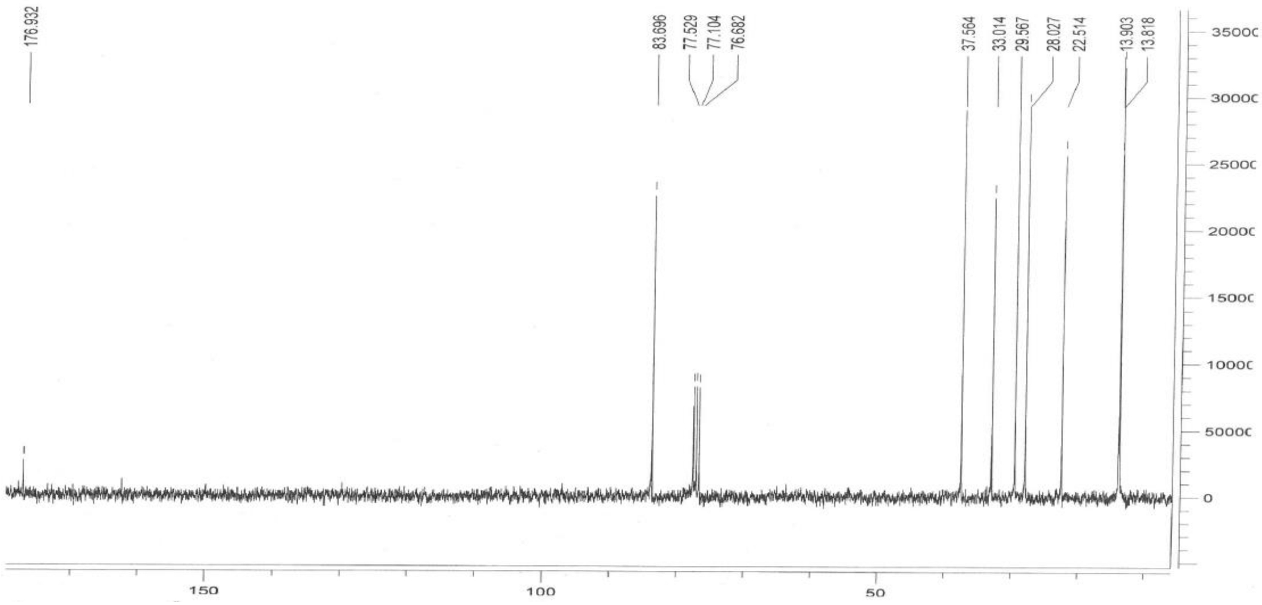

Figure S9. C^{13} NMR spectrum of *cis*-whisky lactone

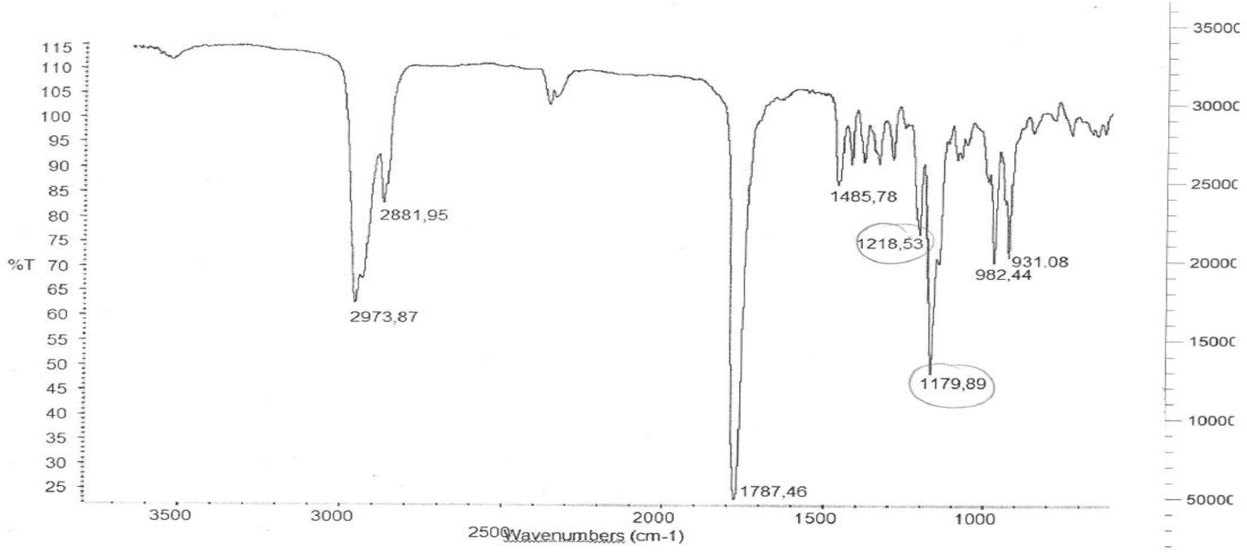

Figure S10. IR spectrum of *cis*-whisky lactone

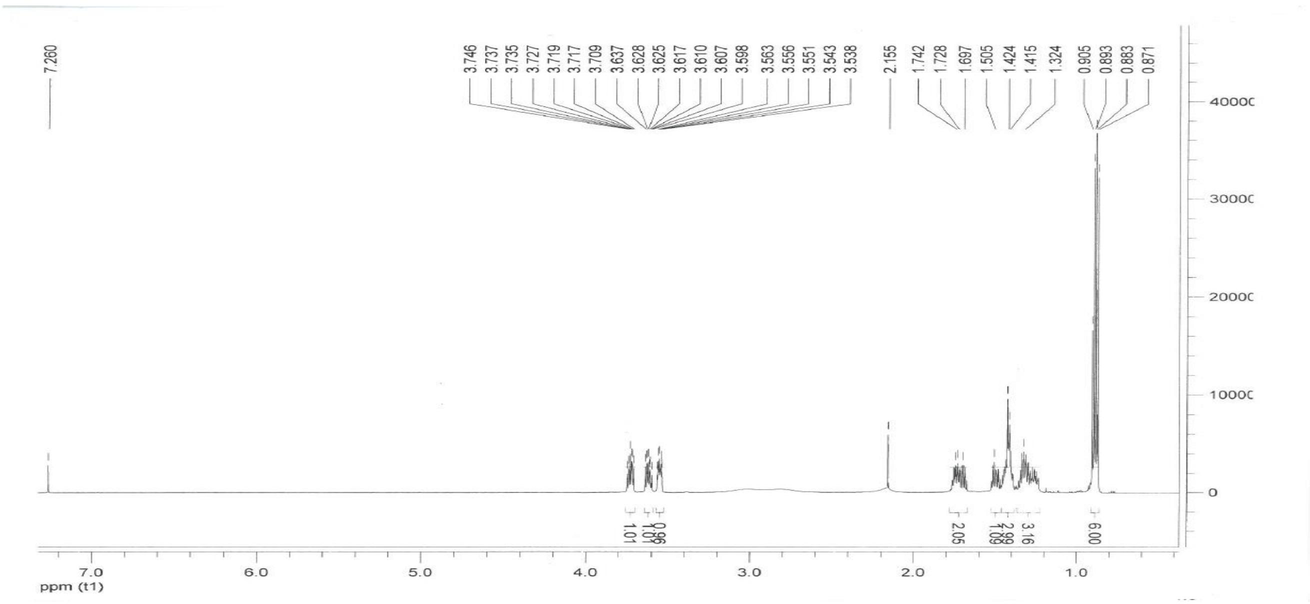

Figure S11. H^1 NMR spectrum of *anti*-3-methyl-octane-1,4-diol

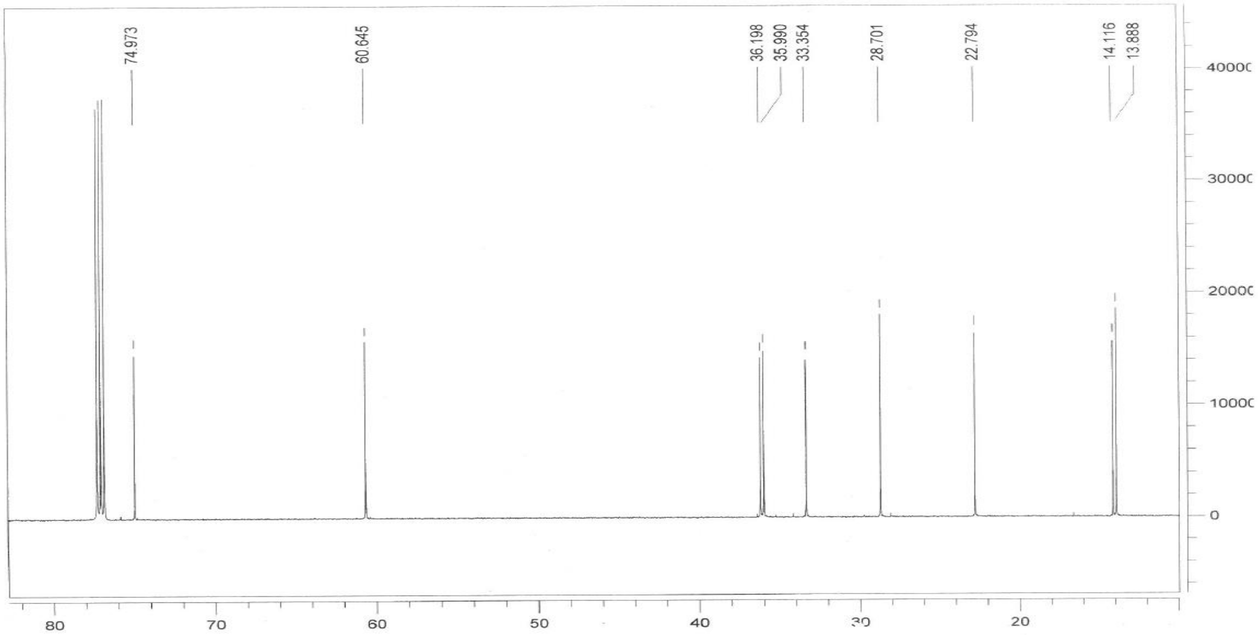

Figure S12. C^{13} NMR spectrum of *anti*-3-methyl-octane-1,4-diol

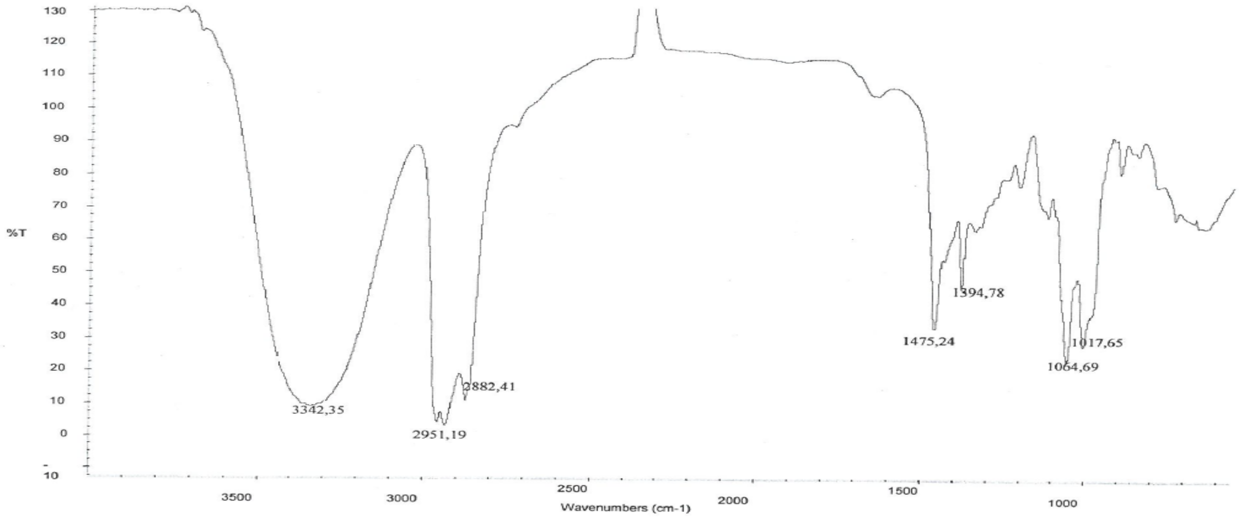

Figure S13. IR spectrum of *anti*-3-methyl-octane-1,4-diol

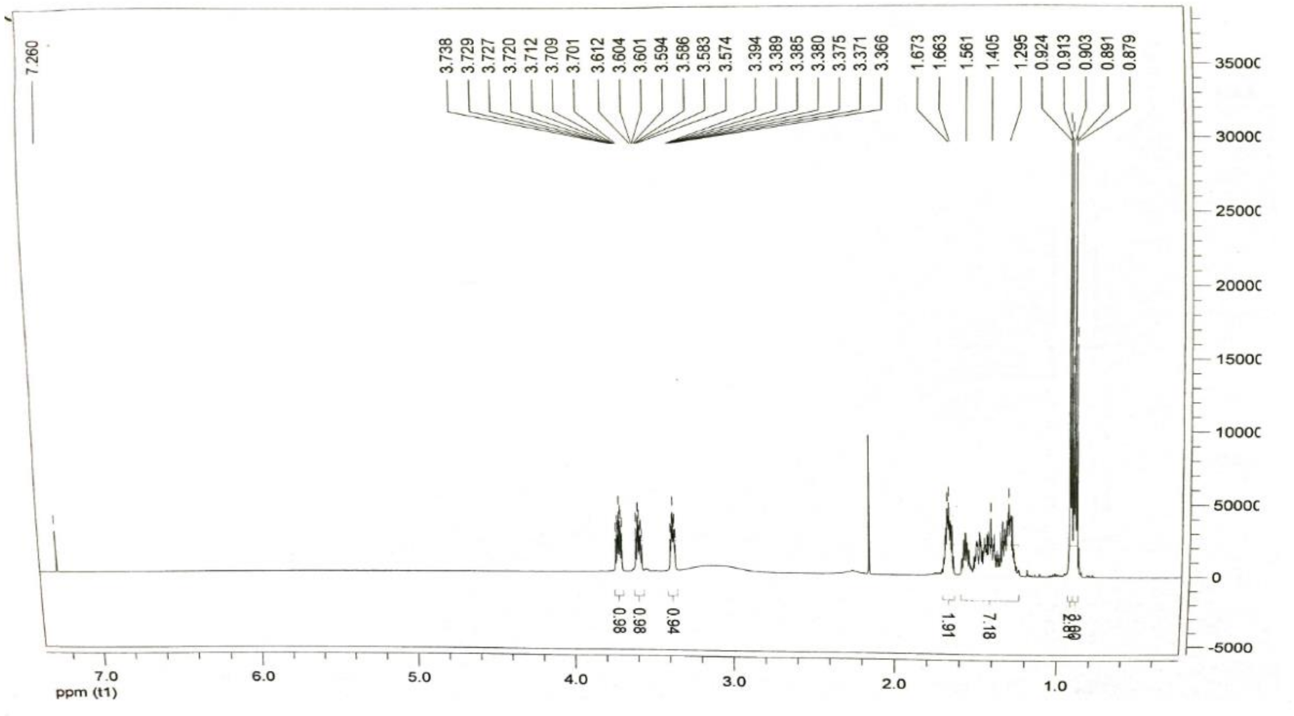

Figure S14. H^1 NMR spectrum *syn*-3-methyl-octane-1,4-diol

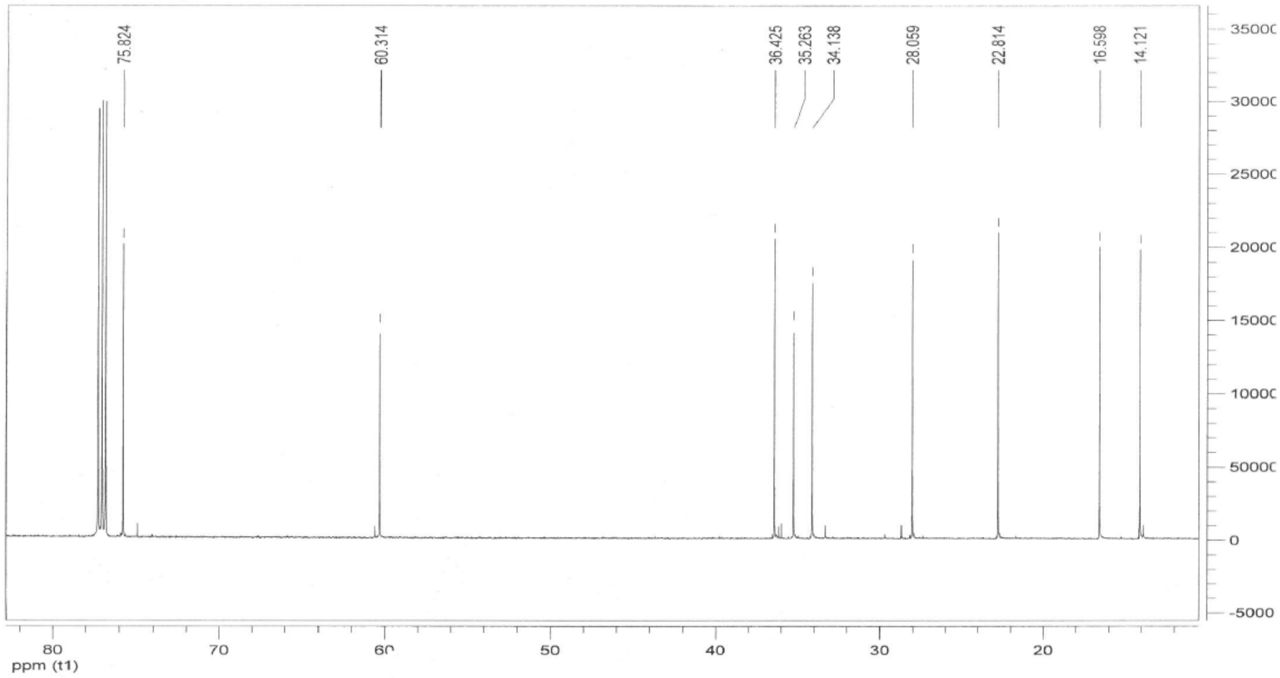

Figure S15. C^{13} NMR spectrum *syn*-3-methyl-octane-1,4-diol

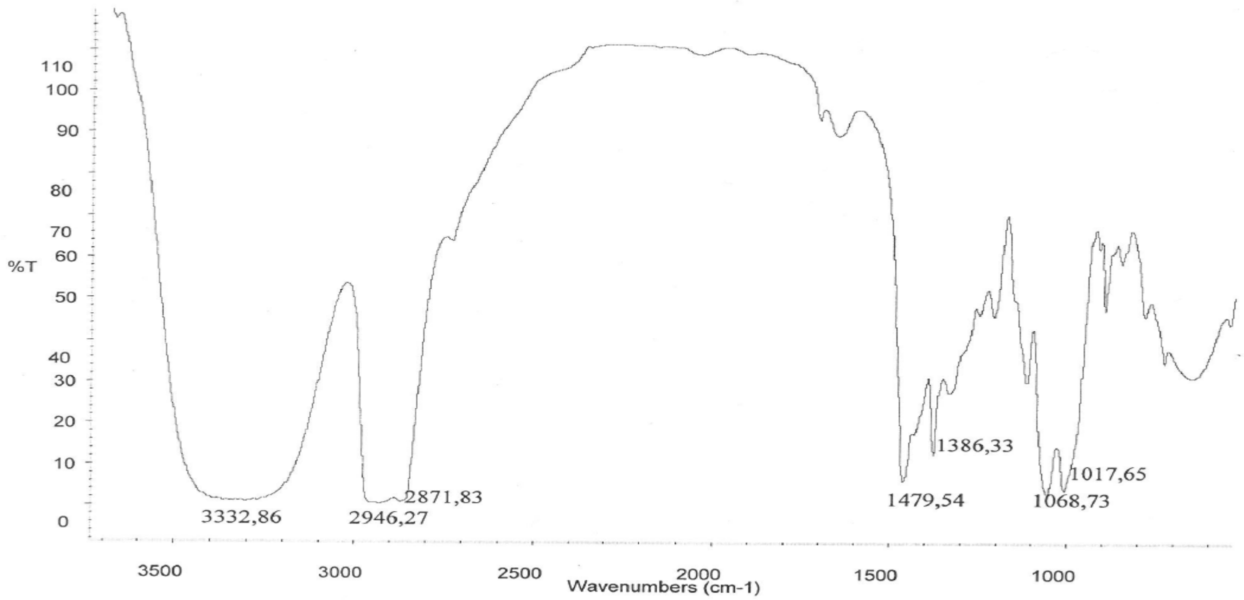

Figure S16. IR spectrum *syn*-3-methyl-octane-1,4-diol
